# Editorial Synthesis for, ‘Parenting Across the Lifespan: Perinatal Mental Health, Infant Feeding, and Child Development’

**DOI:** 10.3390/ejihpe16020027

**Published:** 2026-02-14

**Authors:** Leanne Jackson

**Affiliations:** Department of Psychology, Institute of Population Health, University of Liverpool, Liverpool L69 7ZA, UK; leanne.jackson@liverpool.ac.uk

## 1. Introduction

The first 1001 critical days, spanning from conception to two years postpartum, are a critical window for infant development (Leach, 2017). Beyond this, in a child’s years of dependency (<18; National Health Service [NHS], 2024), they experience various developmental milestones and educational transitions (Cuconato & Walther, 2015; UNICEF, 2025). These transitions and milestones are characterised by increased risks of interpersonal conflict, parent–child attachment disruption, and parental stress (Jones et al., 2021; Laursen et al., 2017). Parental emotional attunement and parenting style during these critical life transitions influence how a child learns to orientate themselves in the external world (Miller et al., 2018; Neel et al., 2018). Indeed, the quality of the parent–child relationship and early year interactions have longstanding impacts on habit formation which endure into adulthood (Kim & Bost, 2023; River et al., 2022). As habits remain relatively stable over time (Ahola et al., 2025), early interventions that adopt a holistic approach to health and wellbeing, particularly ones that recognise the familial influences on child outcomes, are essential.

Several recent developments in legislation in the UK demonstrate a growing commitment to this holistic view of children’s health and wellbeing. In July 2025, the Department for Education (Department for Education, 2025) announced their commitment to improving child development outcomes in children under five, which includes the priorities of financial investment into Best Start family hubs, childcare financial aid, increased Ofsted inspections, and ringfenced funding to promote inclusion in early years educational settings. There are also efforts being made to address childhood poverty (Department for Work and Pensions, 2025). These commitments are being advocated globally (UNICEF, 2019), reflecting broader, family-centred shifts in interventional attitudes. The articles published in the current Special Issue contribute to the growing literature base which identifies the support and wellbeing needs of new parents, with an overarching aim of nurturing positive long-term infant development outcomes.

To summarise the literature included in the current Special Issue, countries spanned Israel (n = 1); Italy (n = 2); United Kingdom (n = 1); Bahrain, Qatar, and Kuwait (n = 1); Portugal (n = 1); Australia (n = 1); and China (n = 1). Findling et al., Guarneri et al., Levante et al., and Xu and Zhang used quantitative, cross-sectional designs; Jackson et al. used a qualitative cross-sectional design; Saidi et al. used a retrospective time-series design on secondary data (spanning from 1990 to 2022); Fuertes et al. used a longitudinal observation design with father–infant dyads (spanning 3, 9, and 12 months postpartum); and Rofe et al. used a within-subjects experimental design. Findling et al., Guarneri et al., and Jackson et al. conducted their studies with mothers only; Fuertes et al. and Rofe et al. conducted their studies with fathers only; Levante et al. and Xu and Zhang conducted their studies with both mothers and fathers; and Saidi et al. investigated the structural determinants of infant mortality by analysing country-level data.

Jackson et al., Levante et al., and Rofe et al. collected primary data via self-selected social media-based recruitment; Findling et al. recruited via mother–child health centres; Fuertes et al. and Guarneri et al. recruited via local hospitals; Saidi et al. conducted a secondary analysis of cross-country data; and Xu and Zhang conducted a secondary analysis on national-level data. A total of 997 pregnant women, 416 postpartum mothers, 114 fathers, and 750 parents (sex and/or gender not clarified) participated across the synthesised articles. Guarneri et al. and Xu and Zhang, 2025, conducted multivariate regression analyses on their data; Findling et al. and Saidi et al. used structural equation modelling; Fuertes et al. used a binary logistic regression; Levante et al. used multiple mediation; Rofe et al. and Xu and Zhang used a one-way repeated measures ANOVA; Xu and Zhang used a moderation analysis; and Jackson et al. used a reflexive thematic analysis.

Xu and Zhang identified parenting stress as a significant negative predictor of parental mental health, whereas objective, subjective, and social support utilisation all had significant positive effects on parental mental health. Other significant predictors of postnatal depression, identified by Guarmeri et al., included prenatal attachment, the couple’s relationship quality, and emotional neglect. In Guarmeri et al.’s model predicting maternal affectivity, emotional abuse and dyadic adjustment were the only significant predictors. These findings were also true across studies which investigated parental stress and parental mental health among the parents of children with additional support needs.

Xu and Zhang found that for the parents of children with a disability, the negative effect of parenting stress on parental mental health was significantly buffered by objective support, but not subjective support. In Levante et al.’s investigation into the parents of children with autism, parenting stress, but not perceived caregiver burden, significantly mediated the relationship between parental resolution and the quality of the parent–infant relationship. In this study, parental resolution was positively correlated with the quality of the parent–child relationship. In Findling et al.’s study on the parents of children With Special Needs and Disabilities (W-SND), the perceived caregiver burden strongly predicted parental burnout, with disability severity further elevating the perceived parenting burden. In the same investigation, a moderation analysis revealed that higher learned resourcefulness was associated with a significantly weaker relationship between the parenting burden and parental burnout, indicating a buffering effect. Findling et al. found that deep emotional work was significantly positively correlated with parental burnout among the mothers of children W-SND. This mirrors Jackson et al.’s qualitative investigation, in which negative feelings were found to be frequently suppressed to maintain the “good mother” identity, generating additional emotional exhaustion. In this qualitative investigation, postpartum mothers described their intense guilt and shame when their lived parenting experience deviated from societal intensive mothering expectations, which were often accompanied by ruminative thoughts.

Across the countries examined and the diverse parenting populations, parenting stress consistently predicted poorer parental mental health. This supports the existing stress-vulnerability frameworks (Duncan et al., 2024) and suggests that they apply robustly across contexts (e.g., parents with disabilities, parents of autistic children, parents of children W-SND). Perceived satisfaction with social support was a key protective factor against poor parental mental health, as was parental resolution, identifying key areas for prioritising interventions, and ringfencing financial investment in 0–5 services, particularly for the parents of children with additional support needs. The evidence from these synthesised works also highlights the importance of considering parental dyads and/or wider family involvement in promoting positive infant outcomes, reflecting a holistic life course approach to health and wellbeing (Hosseini & Ahmadi, 2025).

Interestingly, infant temperament also plays an important role in child health outcomes. In Fuertes et al.’s longitudinal study spanning the first postpartum year, infants who were perceived to be more difficult in temperament and who were less cooperative at three months postpartum were significantly more likely to be prescribed antibiotics when compared with more “cooperative” infants. This suggests that prescribing decisions may be influenced by factors such as the caregiver’s stress or the clinician’s perception, which can be subject to bias, rather than clinical need alone. The integration of decision-making tools, such as digital screening, can help to standardise the process of prescribing medications and thus minimise biases (Clarke et al., 2024). Other potential solutions include providing anticipatory guidance on infant behaviour to new parents and nurturing parental resolve and resources, as identified in the studies of Findling et al. and Levante et al. Such collective efforts may serve to reduce the pathologisation of children’s behaviour.

## 2. Structural and Environmental Determinants

Saidi et al. used 33 years of national health data to demonstrate that health resources have direct effects on infant mortality rates across Bahrain, Qatar, and Kuwait. Specifically, female employment, female educational enrolment, and lower air pollution were all found to significantly reduce infant mortality rates. Similarly, Fuertes et al. found that infants in centre-based daycare were significantly more likely to receive antibiotics than children who were not in centre-based daycare, reflecting an increased exposure to novel pathogens (Houser et al., 2024).

## 3. Intervention Delivery Preference

In Rofe et al., fathers consistently rated intervention programme length as a less important factor than any other programme attributes, emphasising that programme content was significantly more important than facilitator qualities or delivery modality. Of these factors, their decision to engage relied firstly on the parenting program content, followed by the cost, and then the style of participation. The top three preferred parenting programmes in Rofe et al. were all free, had practical activities as the content, and involved face-to-face delivery. No significant differences were observed between individual participation and participation with other fathers or parents.

## 4. Limitations and Future Research Directions

Country-level variability in population characteristics, gender proportions, financial structures, cultural practices, and health policies may limit the generalisability of the findings derived from the synthesised works in the Special Issue. Additional methodological limitations were identified; for example, Saidi et al.’s dataset possessed insufficient statistical power, Fuertes et al.’s laboratory-based observation of dyads lacked ecological validity, and the use of cross-sectional designs, such as in the study by Guarmeri et al., limited the ability to infer causal relationships.

Levante et al. advised that future research should better capture autism severity, Findling et al. recommended capturing the degree of learned resourcefulness, and Jackson et al. recommended routinely recording birth mode; collectively, these can help researchers to better consider experiential nuance when determining parental and child outcomes. Levante et al. also believed that greater representation of autistic girls was essential, and Guarmeri et al. urged an increased representation of fathers in parental research to better acknowledge the role of the broader family unit in infant development and wellbeing outcomes. Greater consideration of potential environmental, institutional (e.g., hygiene practices, infection control policies), and individual (e.g., family health behaviours, prior illness episodes, or cultural attitudes) confounders was also recommended by Fuertes et al., which highlight the need for a greater recognition of the complexity of parental experiences and their relationship with child outcomes.

Fuertes et al., Guarmeri et al., Jackson et al., Levante et al., Rofe et al., and Xu and Zhang sampled parents who were Caucasian, undergraduate-educated or higher, employed, married or cohabiting, exhibited elevated depression and anxiety, and/or resided in areas with strong healthcare infrastructure. This pattern reflects broader inequities in perinatal psychology research related to poor participant representation (Jackson et al., 2021). In response to these limitations, researchers are encouraged to actively diversify their recruitment strategies to better include underserved parent populations, for example by building sustained partnerships within target communities (Fazli et al., 2025) and reducing reliance on social media-based recruitment, which tends to overrepresent socioeconomically privileged groups.

## 5. Conclusions

To summarise the findings presented, a multi-layered picture of early parenting and infant wellbeing can be observed. Parents with extraordinarily high caregiving demands, and those navigating contradictory parenting expectations, may be further strained when structural supports (e.g., flexible employment, safe environments, accessible health services) are inadequate. Similarly, infant behavioural difficulties can increase both parental emotional labour and perceived parenting burden, which can negatively affect lived experiences of parenting.
